# Caregiver-reported profiles of child functioning for children with developmental delays and disabilities 20 months after the onset of the COVID-19 pandemic

**DOI:** 10.3389/frcha.2026.1593822

**Published:** 2026-03-27

**Authors:** Afiqah Yusuf, Nicky Wright, Julie Scorah, Andy Shih, Keiko Shikako, Mayada Elsabbagh

**Affiliations:** 1 Department of Neurology and Neurosurgery, McGill University, Montreal, QC, Canada; 2 Research Institute of the McGill University Health Centre, Montreal, QC, Canada; 3 School of Psychology, Manchester Metropolitan University, Manchester, United Kingdom; 4 Autism Speaks, New York, NY, United States; 5 School of Physical and Occupational Therapy, McGill University, Montreal, QC, Canada

**Keywords:** COVID-19, developmental disabilities, latent class analysis, longitudinal study, parenting self-efficacy, resilience, school access

## Abstract

**Background:**

The COVID-19 pandemic raised concerns about its impact on children with developmental delays and disabilities (DDDs). Early research showed diverse trajectories, with some children experiencing worsening challenges while others demonstrated resilience. In our prior study, conducted 3 months into the pandemic, we identified a resilient group associated with higher parenting self-efficacy and easier access to schooling. As the pandemic persisted, children's functioning may have fluctuated. While adults showed varied trajectories 1 year post-pandemic, little is known about longer-term patterns in children with DDDs. Identifying these patterns nearly 2 years later, along with predictors of resilience, is essential for guiding future interventions.

**Objectives:**

To identify patterns of functioning 20 months post-pandemic onset and examine associations with gender, parenting self-efficacy, and access to schooling.

**Methods:**

As part of the UNICEF-WHO Global Report initiative, we conducted online caregiver surveys across Canada at T1 (June–July 2020) and T2 (November–December 2021). Latent class analysis identified profiles of functioning at T2, and multinomial logistic regression examined associations with T1 predictors.

**Results:**

Among 302 respondents, we identified four profiles:

- Moderate Stability (*n* = 107): Moderate probability of stability (53%–65%), low worsening (7%–26%), and some improvement (18%–39%).
- Worsening (*n* = 80): No improvement and moderate worsening (23%–78%) in mental health, repetitive behaviors, and sleep.
- Very Stable (*n* = 42): 100% probability of stability across domains.
- Mental Health and Sleep Improving (*n* = 73): Improvement in sensory issues (48%), mental health (57%), and sleep (81%), but worsening in repetitive behavior (68%), daily living (50%), and troubling behavior (45%).

Gender, parenting self-efficacy, and access to schooling at T1 significantly predicted profile membership. Girls were more likely to be in favorable profiles. Higher parenting self-efficacy increased the likelihood of belonging to stable or improving profiles. Easier school access at T1 predicted stability or improvement, while difficult access reduced improvement likelihood.

**Conclusion:**

Early-pandemic factors—gender, parenting self-efficacy, and school access—significantly predicted resilience in children with DDDs. These factors may serve as intervention targets to promote long-term stability and recovery.

## Introduction

The COVID-19 pandemic, which commenced in March 2020, brought significant disruptions to daily life worldwide. Public health mandates, designed to curb the outbreak, encompassed the suspension of routine activities like work, school, and social programs, with the aim of enforcing social isolation and distancing. Although there were widespread concerns that both the pandemic and the accompanying public health measures would inflict lasting psychosocial harm on the population, evidence suggests a diverse response to the pandemic with certain sub-groups of individuals exhibiting resilience in the face of adversity (1, 2). Consequently, the pandemic has contributed to a growing body of literature on resilience, highlighting the factors that enable individuals to maintain stability or recover despite prolonged adversity.

A systematic review and meta-analysis of 65 longitudinal studies of children and adults with pre and post-pandemic data from the same participants found only a minor increase in overall mental health symptoms (3). Symptoms stabilized to pre-pandemic levels between May and July 2020, suggesting that the COVID-19 pandemic had only a minimal overall psychosocial impact on the population. Similarly, a systematic review and meta-analysis on Canadian youth found that depressive symptoms worsened minimally from pre-pandemic to wave 1 (March–August 2020) but returned to pre-pandemic levels by wave 2 (September 2021-mid-February 2021) and remained stable to wave 3 (mid-Feb to June 2021) (4). In contrast, anxiety levels increased from wave 1 to wave 3, follow-up data were limited. A recent systematic review and meta-analysis of 21 studies focusing on children and young people with neurodevelopmental conditions also found inconsistent effects of the COVID-19 pandemic on mental health (5).

These findings suggest a variable response to the pandemic, with some individuals experiencing a worsening in outcomes, and others exhibiting resilience—“better-than-expected” outcomes in the face of adversity (6, 7). One large study in the Netherlands identified distinct profiles in anxiety and depression symptoms before and during the pandemic, In this study, individuals with less personal and economic capital were more likely to belong to profiles characterized by high levels of anxiety and depression before and during the pandemic (8). Similarly, a study in Italy analyzed trajectories combining stress, anxiety, and depression among adults. It identified three distinct profiles, with a majority (54%) exhibiting a resilient mental health trajectory characterized by minimal changes in depression, anxiety, and stress (9). Predictors such as age, sex, educational level, employment status, intolerance of uncertainty, cognitive reappraisal, expressive suppression, and fear of COVID-19 were significantly associated with membership in vulnerable mental health classes over the resilient trajectory.

Other studies focusing on adolescents reported similar findings. For example, a study tracking depressive and anxiety symptoms in a representative sample of Hong Kong Chinese individuals aged 15 years and older at three intervals over 18 months found comparable trends (10). They identified four trajectories of symptoms, with a majority exhibiting persistently low levels, labeled “resilient,” while others fell into categories of “chronic distress,” “recovery,” or “delayed distress.” The authors found that those with greater self-efficacy, social support, and financial capacity were more likely to belong to the resilient group. These findings align with other studies that have examined symptom trajectories, reinforcing the idea that a resilient response to the pandemic is a common pattern, with protective factors such as self-efficacy, social support, and financial stability playing a key role in fostering resilience.

The impact of the pandemic on children and youth with developmental delays, disorders, and disabilities (DDDs) was more pronounced due to disruptions in essential support services, including intervention delivery and access to schools, daycares and/or health and social services (citations). One study characterizing profiles of mental health trajectories during the pandemic found 14%–31% of their sample followed a high level of mental health difficulties and were more likely to have special educational needs and/or neurodevelopmental disorders (11). Evidence on broader domains of functioning in youths with DDD's suggests a heterogeneous response to the COVID-19 pandemic. Some domains, like behavioural issues and sleep, showed worsening outcomes, others, such as mental health and autonomy, demonstrated improvement or stability. Socialization and communication outcomes were more variable, reflecting mixed results across studies (12, 13). Qualitative research provides additional context, suggesting that for some families, the slower pace of life and increased quality family time during the pandemic created opportunities to engage in new activities together (14, 15), which may have contributed to positive experiences in certain domains.

In a previous publication from this sample, we surveyed 883 caregivers of children and youth with DDDs across Canada from June to July 2020 (16). We conducted a latent class analysis to examine 12 domains of functioning (including socialization, communication, mental health) among this sample. We identified four profiles: a resilient profile (11% of the sample) characterized by consistent improvements or stability across all domains, a stable profile (46%) showing largely stable patterns of functioning, and two worsening profiles which were combined for analysis (43%) and characterised by declines in functioning across multiple domains.

Longitudinal evidence on mean changes indicates varying patterns of functioning 1 year after the pandemic's onset. For example, one study found that 1 year after the pandemic, adaptive functioning declined among autistic children, yet emotional and behavioral health improved within the same group. In contrast, typically developing children show worsened emotional and behavioural health compared to pre-pandemic levels (17). Another study found worsening emotional and behavioral health among autistic children; however, this decline was not significantly different from that of typically developing children (18). Notably, autistic children with autism spectrum disorders displayed greater interindividual variability in functioning both before and during the COVID-19 pandemic, underscoring the considerable heterogeneity within this population.

We found that the resilient profile of functioning in children with DDDs was more likely in caregivers reporting higher parenting self-efficacy and easier access to schooling for their child (16). Parenting self-efficacy—the caregiver's confidence in helping their child cope—may promote resilience by enhancing the caregiver's ability to access and utilize parenting resources that support their child's functioning. During the pandemic, it is possible that caregivers with high self-efficacy were more likely to have adaptive coping strategies that in turn contributed to the child's or youth's ability to maintain or improve functioning during this period. Similarly, ease of access to schooling was strongly associated with resilience (16), aligning with prior research that identifies education as a vital resource during adversity (19). Facilitating access to schooling—whether remote or in-person—requires equipping educational systems with tools to overcome challenges faced by families to supervise learning. Such efforts can mitigate barriers and enhance resilience in children with DDDs.

In this study, we extended these findings by examining if parenting self-efficacy and access to schooling are associated with longitudinal profiles of functioning, aiming to determine whether these predictors influence resilience beyond the immediate context of the pandemic. Additionally, gender is of interest, as existing reviews of overall mean changes in functioning due to the pandemic in general population samples have identified gender as a moderating variable, with some evidence suggesting that females may be at greater risk (20, 21).

In summary, existing evidence supports the presence of variable profiles of functioning in response to the COVID-19 pandemic. Our previous research identified a resilient profile in children and youth with DDDs 3 months after the onset of the pandemic. In this study we extend these findings to 20 months following the onset of the pandemic, hypothesizing that we will similarly identify a resilient profile. We further examine whether parenting self-efficacy, access to schooling and gender are key predictors of resilience over this extended period.

## Methods

### Design

We utilized data from the Canada site of the Global Report on Developmental Delays, Disorders, and Disabilities survey. This report is an ongoing initiative led by the World Health Organization, UNICEF, and Autism Speaks, aiming to document the experiences of caregivers of children with developmental delays, disorders, and disabilities globally. Following the COVID-19 pandemic, survey questions on pandemic response and related services were added based on disability policy guidance. In Canada, the survey was developed, piloted, and disseminated in collaboration with caregivers, available in English and French. The details of the Global Report can be found in previous publications (16, 22, 23).

We used a longitudinal design on a non-random, convenience sample of caregivers of children with DDDs across Canadian provinces and territories, distributed online via research and parent networks, using a crowdsourcing sampling strategy, including social media platforms and mail lists of partner organizations. Data collection occurred at two timepoints: (1) T1 from June to July 2020, and (2) T2 at 20 months post-pandemic onset in November–December 2021. Caregivers who participated at T1 were re-contacted and invited to participate again at T2, enabling longitudinal follow-up. Participants received a $15 incentive for each wave of participation.

The second wave of data collection occurred amid fluctuating public health measures across Canada (24, 25). In summer 2021, declining COVID-19 infection rates led provinces to ease restrictions at different rates. By July, Alberta and Saskatchewan had lifted most public health measures, while others proceeded more cautiously. Ontario and Quebec allowed limited indoor gatherings, dining, and other activities under capacity restrictions. By fall 2021, many provinces implemented vaccine mandates for employees in hospitals and other enterprises, while nonessential businesses like restaurants and theaters required proof of vaccination. The federal government also mandated vaccination for air and train travel. Schools resumed in-person learning in September 2021, with masking requirements in most provinces except Alberta and Saskatchewan.

As the Delta variant surged in fall 2021, Alberta, Saskatchewan, and British Columbia faced significant pressures, while the Atlantic provinces and central Canada saw smaller waves. By October, many provinces introduced vaccination passports for accessing nonessential establishments. In November, Quebec relaxed certain restrictions, such as lifting capacity limits in public venues and ending remote work recommendations. However, by mid-December, the emergence of the Omicron variant triggered a fifth wave, leading to renewed restrictions, gathering size limits, extended school closures until mid-January 2022, and a shift from PCR testing to rapid antigen tests and wastewater surveillance.

This evolving public health landscape provided critical context for interpreting caregivers' experiences at the second data collection point, highlighting the dynamic nature of the pandemic's impact on families of children with DDDs.

### Participants

A total of 883 caregivers completed the initial survey and 302 caregivers completed the follow-up survey. The demographic characteristics of the sample are reported in the results section (Table 1).

**Table 1:** Demographics of the survey respondents at time 1 (*n* = 883) and the analysed sample who provided follow-up data (*n* = 302).

Variables	T1 sample (*n* = 883)		T2 follow-up sample (*n* = 302)	
Sociodemographic				
Relationship to person with a disability, *n* (%)				
Biological mother	533	(61.3)	204	(67.8)
Biological father	236	(27.2)	62	(20.6)
Other	100	(11.5)	35	(11.6)
Household status, *n* (%)				
Two-parent household	701	(80.9)	266	(88.1)
Single-parent household	158	(18.2)	35	(11.6)
Other	7	(0.8)	0	(.3)
Annual household income, *n* (%)				
≤$39,999	106	(12.3)	40	(13.4)
≥$40,000	755	(87.7)	266	(88.1)
Education of respondent, *n* (%)				
High school or less	76	(8.8)	29	(9.8)
Undergraduate degree or diploma	544	(63.1)	188	(63.3)
Higher education or professional degree	216	(25.1)	70	(23.6)
Other	25	(2.9)	10	(3.4)
Ethnicity of respondent, *n* (%)				
Indigenous	214	(24.7)	90	(30.0)
White	537	(61.9)	179	(59.7)
Asian	46	(5.0)	19	(6.3)
Black	28	(3.2)	3	(1.0)
Other	42	(4.8)	9	(3.0)
Age of child/youth with a disability in years, M (SD)	9.4	(5.7)	9.5	(5.2)
Gender of child/youth with a disability, *n* (%)				
Male	502	(57.8)	170	(56.7)
Female	365	(42.1)	130	(43.3)
Other	1	(0.1)	0	
Diagnoses of child/youth, *n* (%)				
ASD/ID only	168	(19.2)	70	(23.2)
ASD/ID plus other diagnoses	322	(36.9)	86	(28.5)
Diagnoses other than ASD/ID, e.g., troubles with mobility, anxiety, and epilepsy	383	(43.9)	146	(48.3)

### Measures

Specific survey items that were the focus of the current study were as follows [also detailed in Yusuf et al. (16)]:

- Parenting self-efficacy in the context of the COVID-19 pandemic was measured using the question “In general, how confident are you that you can help your child with a disability cope with during the pandemic?”. The response option to this question is a five-point scale from “Not at all confident” to “Extremely confident”. For analysis, parenting self-efficacy was treated as a linear continuous ordinal variable, with higher scores indicating greater perceived confidence in the parenting role.
- Ease in accessing schooling was measured using the item “Getting daycare, preschool or school for your child” under the question stem “During the pandemic, how easy has it been to maintain help and support for your family?”. A five-point response option to this item ranged from “Very easy” to “Very difficult”. Responses were recoded into a three-category variable (somewhat easy/very easy, neither easy nor difficult, and somewhat difficult/very difficult), with the neutral category used as the reference group.
- Gender—caregivers reported on their child's gender and treated as a binary categorical variable (female vs. male) in analyses.

Single-item measures were used to minimise respondent burden in a large, national, rapid-response survey conducted during the COVID-19 pandemic, consistent with prior analyses from this cohort (16).

The outcome variable, child functioning was assessed using the question stem, “During the pandemic, did you notice changes in your child in any of the following areas?” Caregivers were to rate either “Worsened,” “Stable,” or “Improved” on each of the 12 domains of child functioning listed below (see Table 2).

**Table 2:** Conditional probabilities for each of the 12 items by latent class found at follow-up 20 months following COVID-19 onset.

Domain	Latent Class 1 *n* = 42 “Very Stable”			Latent Class 2 *n* = 80 “Worsening”			Latent Class 3 *n* = 107 “Moderate Stability”			Latent Class 4 *n* = 73 “Mental Health and Sleep Improving”		
	Worsened	No change	Improved	Worsened	No change	Improved	Worsened	No change	Improved	Worsened	No change	Improved
Troubling behaviour	0.00	1.00	0.00	0.53	0.47	0.00	0.22	0.57	0.22	0.45	0.55	0.00
Daily living	0.00	0.86	0.14	0.28	0.66	0.06	0.23	0.41	0.36	0.50	0.50	0.00
Health problems	0.00	0.95	0.05	0.23	0.77	0.00	0.16	0.59	0.25	0.08	0.83	0.09
Mental health	0.00	1.00	0.00	0.78	0.22	0.00	0.09	0.60	0.30	0.00	0.43	0.57
Sleep	0.05	0.93	0.02	0.61	0.39	0.00	0.26	0.44	0.31	0.00	0.19	0.81
Diet/eating difficulties	0.01	0.99	0.00	0.49	0.47	0.03	0.21	0.41	0.39	0.00	0.78	0.22
Social interaction difficulties	0.00	1.00	0.00	0.50	0.50	0.00	0.19	0.47	0.35	0.36	0.65	0.00
Repetitive behaviour	0.02	0.98	0.00	0.65	0.35	0.00	0.07	0.66	0.27	0.68	0.28	0.04
Communication difficulties	0.00	0.96	0.04	0.44	0.52	0.04	0.20	0.43	0.37	0.23	0.77	0.00
Safety concerns	0.00	0.96	0.04	0.25	0.71	0.04	0.21	0.58	0.20	0.08	0.65	0.27
Sensory issues	0.00	1.00	0.00	0.50	0.50	0.00	0.21	0.61	0.18	0.15	0.37	0.48
Education	0.05	0.95	0.00	0.46	0.45	0.09	0.15	0.54	0.31	0.08	0.76	0.16

### Statistical analysis

Descriptive statistics were run to examine group-level statistics on the variables of interest. We compared non-respondents with respondents at follow-up on child age, gender, family income, single-parent status, T1 parenting self-efficacy and T1 access to schooling.

Latent class analysis (LCA) was used to create groups of children who showed different profiles of functioning in the pandemic. LCA, a type of mixture modelling, assumes that an underlying categorical variable explains the relationships among item responses, and uses response patterns in the data to identify profiles, or latent classes. The approach is “person-centred” using patterns of response to create groups of individuals, as opposed to factor analysis which is “variable-centred” and groups items together. A series of LCAs were run to examine the underlying number of latent groups on the 12 functioning questions using Mplus version 8 (26). A series of models with different numbers of classes (e.g., two-class, three-class) were estimated and the absolute and relative fit indices of these models were compared. The Akaike's Information Criteria (AIC), Bayesian information criterion (BIC), the Sample-adjusted Bayesian Information Criterion (SBIC) and the Vuong-Lo-Mendel-Rubin (LMR) test statistics were used as statistical criteria to compare models to identify the optimal number of groups to retain (27). Lower AIC, BIC and SBIC values in model-to-model comparisons reflected a better fit. A nonsignificant chi-square value (*p* < .05) for the LMR statistic suggests that a model with one fewer class is preferred. Further, average posterior probabilities and entropy values equal to or greater than .80 indicate clear classification and greater power to predict class membership (28).

We then extracted the class membership groupings and used this as the dependant variable in a multimonial logistic regression in SPSS version 25 (29) to examine associations between class membership and the hypothesised relevant variables. Multinomial regression is an extension of binomial logistic regression which allows for more categories. Separate regression models were run for parenting self-efficacy, ease in access to schooling, and gender.

## Results

### Descriptives

Table 1 describes the demographics of the sample who responded to the survey at both timepoints (*n* = 302). Most respondents were a mother to a person with disability, were in a two-parent household, were White, and reported an annual household income of more than or equal to $40,000 CAD. The respondent's child with disability was on average 9.5 years old (SD = 5.2) and was approximately evenly split between males and females. More than half of the respondents reported a diagnosis of ASD or Intellectual Disability (ID) in their child. Twenty three percent of the sample had an ASD/ID diagnosis only, and twenty nine percent reported a diagnosis of ASD/ID plus at least one other diagnosis in addition to the ASD/ID diagnosis. The most common co-occurring diagnoses in the children/youths with ASD/ID were sleep disorders (37.2%), anxiety (31.4%), gastrointestinal difficulties (27.9%), and epilepsy (24.4%). The remaining 33% of respondents reported no ASD/ID but other conditions, with the most reported diagnoses as follows: troubles with mobility (26.7%), allergies (22.6%), epilepsy (19.2%), and vision/hearing problems (16.4%).

Those who responded to the follow up survey were more likely to be mothers [*X*^2^ = 10.527 (2) *p* = .005], single parents [*X*^2^ = 14.353 (1) *p* < .001], less likely to have a child with an autism/ID plus other diagnoses [*X*^2^ = 18.140 (2) *p* < .001] compared to the other diagnosis groups, and less likely to be of black ethnicity [*X*^2^ = 14.200 (4) *p* < .001]. There was no association between responding to the follow-up and low income (*p* = .274) and parental education (*p* = .717) nor with the three predictors included in this analysis: child gender (*p* = .311), school access (*p* = .321) or parental coping (*p* = .800).

### Latent class analysis

We conducted latent class analysis to examine the underlying number of latent classes on care- giver-reported changes of the 12 functioning domains at follow-up 20 months following the COVID-19 pandemic onset. As shown in Supplementary Table S1, the absolute fit statistics decreased from the three-class model (BIC = 6,646.33, SBIC = 6,411.64, AIC = 6,371.5) to the four-class model (BIC = 6,550.37; SBIC = 6,235.03; AIC = 6,182.38) and increased again for the five-class model (BIC = 6,625.54, SBIC = 6,232.27, AIC = 6,164.62). In addition, the V-LMR test statistic fell out of significance for the five-class model (*p* = .772). Taken together, the four-class model best represented the data based on the absolute and V-LMR statistics.

**Figure 1:**
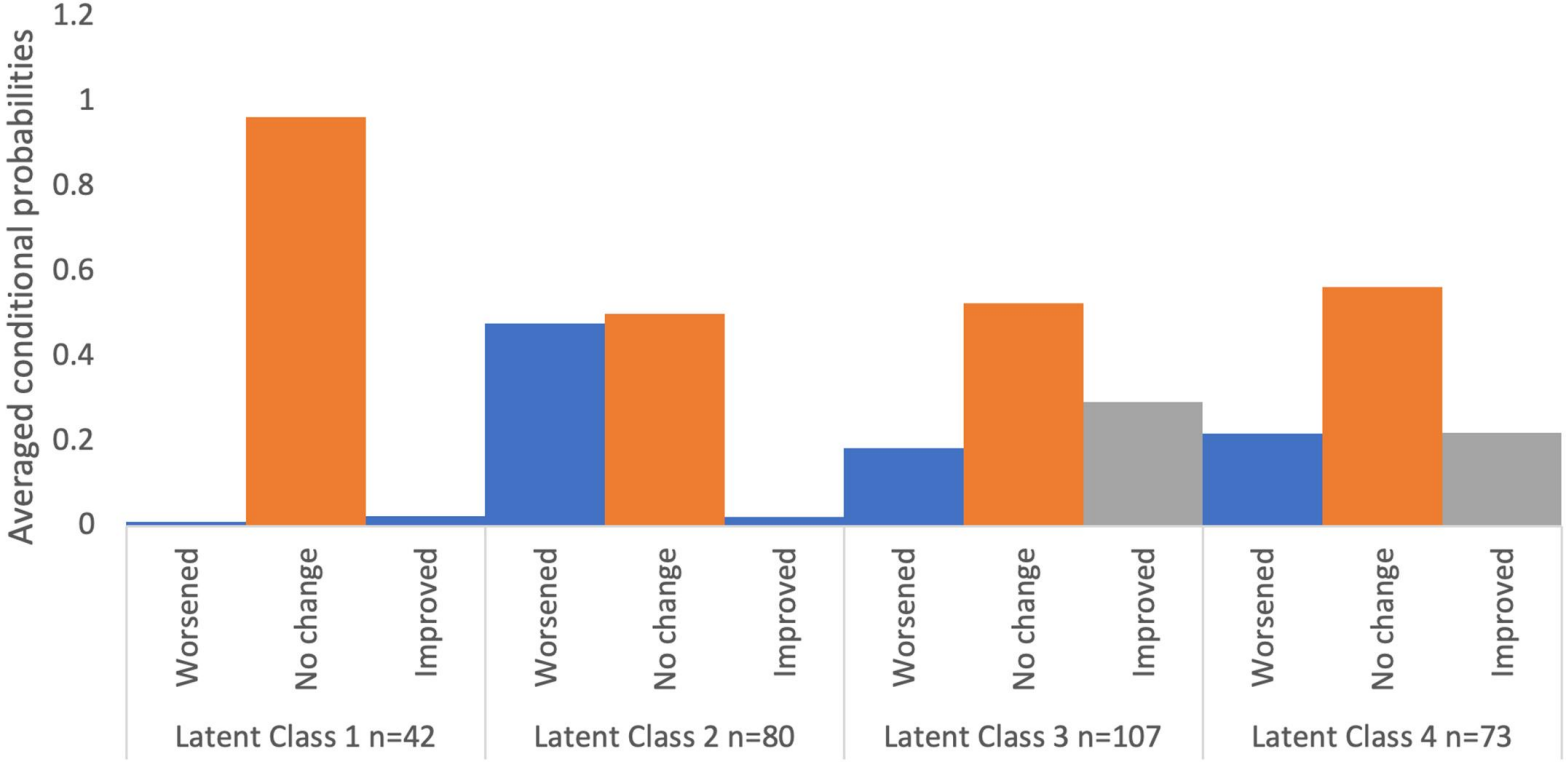
Averaged conditional probabilities for each of the four classes at follow-up 20 months following the COVID-19 onset.

Classification quality for the four-class solution was high, with average posterior probabilities for most-likely class membership ranging from 0.93 to 0.98 across classes (Supplementary Table S2). The entropy value was.91. The conditional probabilities for the items for each latent class are shown in Table 2 and the averaged conditional probabilities for each of the four classes are visualized in Figure 1.

Class 1 (*n* = 42) showed virtually 100% chance of no change across all domains, with zero chance of worsening or improving. We labeled this class as “Very Stable”.

Class 2 (*n* = 80) also showed consistently zero chance of improving across all domains but there was also a moderate chance of worsening (range 23%–78%, average = 48%) across all domains, particularly in mental health (78%), repetitive behaviour (65%), and sleep (61%). “No change” was reported with moderate probabilities as well (range 22%–77%, average = 50%), especially in health problems (77%), safety concerns (71%), and daily living (66%). This class was named “Worsening”.

Classes 3 and 4 both show similar averaged conditional probabilities of worsening (average of 18% and 22%, respectively), no change (53% and 56%), and improvement (22% and 29%). Class 3 (*n* = 107) is characterized by a moderate likelihood of no change (range 54%–65%) in troubling behaviour, health problems, mental health, repetitive behaviour, safety, sensory, and education. There is a small chance of improvement (range 18%–39%) and worsening (range 7%–26%) across all domains. Class 4 (*n* = 73) shows moderate to high chance of improvement in sensory issues (48%), mental health (57%), and sleep (81%) but also exhibits worsening in troubling behaviour (45%), daily living (50%), and repetitive behaviour (68%). There is moderate to high chance of stability (range 50%–83%) in other areas such as daily living, troubling behaviour, health problems, diet/eating, social interaction, communication, safety, and education. Thus, we termed Class 3 as “Moderate stability”, and Class 4 “Mental Health and Sleep Improving”.

Table 3 presents a cross-tabulation of caregiver class distributions from T1 (previously published, Supplementary Tables S1, S2) to T2. Due to the small cell sizes, statistical tests to compare proportions were deemed inappropriate; thus, we focus on describing the proportions. At T1, 80% of caregivers in the “Stable” class, characterized by a 50% probability of no change across most domains 3 months post-COVID-19 onset, transitioned to either the “Mental Health and Sleep Improving” or “Moderate Stability” classes at the 20-month follow-up. Youth in the “Resilient” class, who had a zero probability of worsening and an equal chance of improvement or stability, predominantly shifted to the “Moderate Stability” class. The “Somewhat Worsening” class exhibited a varied transition pattern, with roughly one-third moving to the “Worsening” class, one-third to the “Very Stable” class, and the remaining third to either the “Mental Health and Sleep Improving” or “Moderate Stability” classes. Lastly, youth from the “Clear Worsening” class at T1 mostly remained in the “Worsening” class at follow-up.

**Table 3:** Cross-tabulation of latent classes from T1 to T2.

Classes at T2	Classes at T1				
	Stable	Resilient	Somewhat worsening	Clear worsening	Total
Very stable	13 (8.4%)	0 (0%)	26 (31.3%)	3 (6.7%)	42
Mental health and sleep improving	57 (36.8%)	2 (10.5%)	13 (15.7%)	1 (2.2%)	73
Moderate stability	67 (43.2%)	16 (84.2%)	16 (19.3%)	8 (17.8%)	107
Worsening	18 (11.6%)	1 (5.3%)	28 (33.4%)	33 (73.3%)	80
Total	155 (100%)	19 (100%)	83 (100%)	45 (100%)	302

We performed a multinominal logistic regression to examine the association between candidate predictors and profile membership. Table 4 details the results of the regressions examining the extent to which child gender, parenting self-efficacy, and ease in accessing schooling was associated with the likelihood of being classified into each of the participant profiles.

**Table 4:** Multinominal logistic regression to examine association between child gender, parenting self-efficacy, and access to schooling with class membership.

Candidate predictors at T1	Class 1 “Very Stable” (*n* = 42) vs. Class 2 “Worsening” (*n* = 80)	Class 3 “Moderate Stability” (*n* = 107) vs. Class 2 “Worsening” (*n* = 80)	Class 4 “Mental Health and Sleep Improving” (*n* = 73) vs. Class 2 “Worsening” (*n* = 80)
	OR (90% CI)	OR (90% CI)	OR (90% CI)
Female gender (*n* = 130) vs. male (*n* = 170)	3.34 (1.52, 7.34)**	2.25 (1.20, 4.21)*	3.09 (1.56, 6.10)**
Confidence in helping child cope	1.90 (1.33, 2.70)***	1.90 (1.43, 2.53)***	1.74 (1.29, 2.38)***
Schooling: Somewhat easy or very easy (*n* = 76) vs. neither easy nor difficult *n* = 82)	2.46 (.64, 9.49)	3.71 (1.31, 10.54)**	3.67 (1.27, 10.63)*
Schooling: Somewhat or very difficult (*n* = 141) vs. neither easy nor difficult *n* = 82)	.94 (.37, 2.372)	.53 (.27, 1.01)	.27 (.12,.59)**

*** *p* < .001.

** *p* < .01.

* *p* < .05.

As shown in Table 4 child gender, parenting self-efficacy, and ease of accessing schooling at T1 were predictive of membership in the Very Stable, Moderately Stable, Mental Health and Sleep Improving classes compared to the Worsening profile at follow-up, 20 months following the COVID-19 onset. Specifically, female children and youths were at least 2.25 times more likely to be in the Moderately Stable, Very Stable, or Mental Health and Sleep Improving profiles than in the Worsening profile, compared to their male counterparts. Children and youths whose caregivers reported higher parenting self-efficacy at T1 were more likely to belong to the Very Stable (OR = 1.90, 90% CI: 1.33–2.70), Moderately Stable (OR = 1.90, 90% CI: 1.43–2.53), or Mental Health and Sleep Improving (OR = 1.74, 90% CI: 1.29–2.38) profiles at T2 compared to the Worsening profile. Additionally, children and youths whose caregivers reported somewhat easy or very easy access to schooling at T1 were significantly more likely to belong to the Moderately Stable (OR = 3.71, 90% CI: 1.31–10.54) or Mental Health and Sleep Improving (OR = 3.67, 90% CI: 1.27–10.63) profiles at T2 compared to those whose access to schooling was rated as neither easy nor difficult. Conversely, those who experienced somewhat or very difficult access to schooling at T1 were significantly less likely to belong to the Mental Health and Sleep Improving profile (OR = 0.27, 90% CI: 0.12–0.59) than the Worsening profile at T2.

## Discussion

The COVID-19 pandemic brought about unprecedented changes in daily life, with widespread concerns about its potential psychosocial impact on individuals, especially children and youth with developmental delays and disabilities (DDDs). We previously established three distinct profiles of functioning in youth with DDDs immediately post-lockdown: 43% belonged to a profile characterized by worsening in functioning, 46% showed a fairly stable response, and 11% exhibited resilience with evidence of clear improvements across domains. At 20 months post-pandemic onset, during the fifth wave of the COVID-19 pandemic when many restrictions had lifted but some remained, we identified four latent classes of functioning. The majority of participants belonged to profiles reflecting either completely stable functioning (Very Stable, 14%) or variable but largely stable functioning (Moderate Stability, 35%). A smaller proportion (26%) belonged to a Worsening profile, indicating a decline in functioning across most domains, although this group was smaller than the 43% identified previously in the immediate aftermath of the pandemic onset. Notably, unlike our earlier findings, we did not identify a profile reflecting clear improvement across all domains. Instead, we identified a Mental Health and Sleep Improving profile (24%) characterized by domain-specific resilience, particularly in mental health and sleep, with moderate stability across other domains.

When examining transitions from T1 profiles, we observed substantial continuity, with most participants from the earlier Stable profile transitioning into the Moderate Stability or Mental Health and Sleep Improving profiles at T2. Participants from the Resilient profile at baseline also predominantly transitioned into the Moderate Stability profile, suggesting sustained but moderate resilience over time. Conversely, participants in the Clear Worsening profile at T1 largely remained in the Worsening profile at T2, reflecting the persistence of challenges for this subgroup. These transition patterns should be interpreted in light of the broader pandemic context at the time of the second assessment, which occurred during the fifth wave of COVID-19, when many public health restrictions had been lifted but uncertainty and disruptions persisted. As such, the observed profiles likely reflect adaptation to prolonged and evolving pandemic conditions rather than a return to pre-pandemic functioning.

Our findings indicate that child gender, parenting self-efficacy, and ease of access to schooling early in the pandemic significantly predicted membership in favorable functioning profiles 20 months post-pandemic onset. Female children and youths were consistently more likely to belong to the Very Stable, Moderate Stability, or Mental Health and Sleep Improving profiles compared to the Worsening profile (ORs ranging from 2.25 to 3.34). Caregivers reporting higher parenting self-efficacy were also more likely to have children in these favorable profiles, with odds ratios ranging from 1.74 to 1.90. Furthermore, children whose caregivers reported somewhat easy or very easy access to schooling were significantly more likely to belong to the Moderate Stability (OR = 3.71, 90% CI: 1.31–10.54) or Mental Health and Sleep Improving (OR = 3.67, 90% CI: 1.27–10.63) profiles. In contrast, children experiencing somewhat or very difficult access to schooling were significantly less likely to belong to the Mental Health and Sleep Improving profile (OR = 0.27, 90% CI: 0.12–0.59) compared to the Worsening profile. These associations should be interpreted with caution given the reliance on caregiver-reported data and the use of a non-random convenience sample, which may introduce reporting and selection biases.

When examining the profiles, the study offers insights into how we can infer resilience from the patterns of change in functioning. Resilience is often understood as the ability to bounce back from adversity and maintain or improve one's well-being (6, 7, 30, 31). In this context, resilience is evident not only in stability but also improvement in functioning during the pandemic. Most participants in our sample were characterized by either stability or improvement across multiple domains of functioning, with a quarter of the sample belonging to a profile showing consistent worsening across domains. In particular, the “Very Stable” group showed almost certain probability of stability across all 12 domains of functioning. Two other groups, while showing some chance of worsening in certain domains, also showed stability and improvement in others. In particular, one group showed a moderate to high probability of improvement in mental health, sleep and sensory issues.

Our results are consistent with other studies that examined overall change in multiple domains of functioning and found different patterns of change (17, 32). For example, Siracusano et al. (32) found no significant worsening on autistic symptoms, adaptive functioning, behavioural problems, and repetitive behaviour among autistic children in May–July 2020 compared to pre-pandemic scores, while Pokoski et al. (17) found significant declines in the communication scores of young children with autism in January–July 2021, while daily living skills, socialization skills, and behavioral and emotional health improved compared to pre-pandemic levels. This finding of most children showing some resilience is consistent with the overall small effects on mental health found in previous studies (5).

The proportion of youth belonging to a worsening profile has changed from what was observed immediately post-lockdown (16) to the present. At T1, 43% of the sample exhibited worsening profiles, while 26% showed a somewhat worsening profile at T2. This shift raises questions about the long-term impact of the pandemic. Studies have consistently identified four trajectories of anxiety and depressive symptoms in the 1–1.5 years following the onset of the COVID-19 pandemic (10, 33): most were in a “Resilient” group characterized by stable, low anxiety and depressive symptoms throughout the period studied. Another group (8%–12%) demonstrated a “Recovery” trajectory where symptoms decreased by March–August 2021. The remaining groups (∼15%) either had persistently high anxiety and depressive symptoms or reported an increase in symptoms throughout the period. Our finding of larger numbers of youths with developmental delays, disabilities, and disorders belonging to profiles characterised by worsening is consistent with these findings.

The study highlights the crucial role of two specific factors in fostering resilience among this population: parenting self-efficacy and ease of access to school. The evidence suggests that caregivers who had higher levels of confidence in their ability to support their children during the pandemic (parenting self-efficacy) and those who faced fewer barriers in terms of providing education for their children were more likely to have children exhibiting resilience in various domains of functioning. Furthermore, what's noteworthy is that these protective effects continued to persist over 18 months until the follow-up assessment in November–December 2021. This finding underscores the enduring impact of these factors on the well-being and adaptability of children and youth with DDDs.

The findings from this study have significant implications for future research and support systems for children and youth with DDDs, especially in times of crisis such as a pandemic. Understanding the changing trajectories of functioning and the factors that contribute to resilience can guide the development of targeted interventions and resources. Taken together, these findings highlight both the heterogeneity of responses among children and youth with DDDs and the importance of situating resilience within specific methodological, contextual, and structural constraints.

## Limitations

Our study is limited by the absence of a static reference point, as we only reported changes perceived by caregivers over the last 3 months at both timepoints. This limitation arises from the lack of T1 measurements or initial assessments that could serve as a comparative reference. Without these, it becomes challenging to quantify the actual magnitude of changes or to discern whether the observed changes are part of a longer-term trend or a short-term fluctuation. Additionally, caregiver perceptions can be subjective and influenced by various factors such as stress, recall bias, and individual expectations. Therefore, the absence of a concrete T1 presents a limitation to our findings and the ability to make definitive conclusions about the nature and extent of changes over time. Future studies could benefit from incorporating objective measures or assessments from multiple sources to enhance the validity of findings. There was also substantial attrition at the follow-up survey (with 34% of the original sample responding). Whilst non-response was associated with some demographics and child diagnostic group, it was not associated with income/education nor any of the predictors examined in the analysis. Although differential attrition raises the possibility of selection bias, adjustment for these covariates in the multinomial regression models was not feasible due to very small cell sizes within several latent class–covariate combinations, which would have resulted in unstable parameter estimates. However, the absence of associations between attrition and the predictors of interest suggests that any remaining bias is more likely to affect estimates of class prevalence rather than associations between predictors and latent class membership. The use of single-item measures may have introduced greater measurement error compared to multi-item scales, which may have attenuated observed associations and should be considered when interpreting effect sizes.

In addition, the generalizability of these findings may be limited by the use of a non-random, convenience sample recruited online through caregiver networks and social media. Participants were caregivers of children with DDDs residing in Canada who had access to digital platforms and were engaged with support or research networks; therefore, the findings may not fully represent families facing greater structural barriers or those in different sociocultural or healthcare contexts. Caution is warranted when extending these results to other populations or settings.

Moreover, the study's reliance on data collected over 20 months limits our understanding of the longer-term effects of the pandemic on this population. It would be valuable for future research to extend the follow-up period to examine profiles of functioning and factors influencing resilience over a more extended duration.

## Conclusion

In conclusion, the study contributes to our understanding of resilience in children and youth with DDDs during the COVID-19 pandemic. It highlights the importance of stability and improvement in functioning, the evolving landscape of challenges, and the enduring impact of factors like parenting self-efficacy and access to education. These insights should inform future research and support systems aimed at promoting the resilience of this population in the face of adversity.

## Data Availability

The datasets presented in this article are not readily available because participants did not provide informed consent for broad sharing of the data collected. Requests to access the datasets should be directed to the corresponding author.
