# Supplementary material for: Caregiver-reported profiles of child functioning for children with developmental delays and disabilities 20 months after the onset of the COVID-19 pandemic
Source: Front Child Adolesc Psychiatry. 2026 Mar 27;5:1593822. doi: 10.3389/frcha.2026.1593822 (PMC13066226; doi:10.3389/frcha.2026.1593822)
Supplement: Supplementary file 1 [file Table1.docx]

**SUPPLEMENTARY MATERIALS**

Table S1: Full fit indices and comparisons for 2-5 class solution models

| **Number of classes** | **AIC** | **BIC** | **SBIC** | **VLMR p-value (k–1 vs k)** | **Entropy** |
| --- | --- | --- | --- | --- | --- |
| 2 | 6547.58 | 6768.47 | 6583.14 | < .001 | 0.79 |
| 3 | 6371.52 | 6646.33 | 6411.64 | < .001 | 0.87 |
| **4** | **6182.38** | **6550.37** | **6235.03** | **0.031** | **0.91** |
| 5 | 6164.62 | 6625.54 | 6232.27 | 0.772 | 0.92 |

Table S2: Average posterior probabilities by class

| **Assigned class** | **P(Class 1)** | **P(Class 2)** | **P(Class 3)** | **P(Class 4)** | **Class n** |
| --- | --- | --- | --- | --- | --- |
| Class 1 | **0.979** | 0.002 | 0.019 | 0.001 | 42 |
| Class 2 | 0.003 | **0.955** | 0.034 | 0.007 | 81 |
| Class 3 | 0.000 | 0.010 | **0.949** | 0.041 | 107 |
| Class 4 | 0.000 | 0.002 | 0.070 | **0.928** | 73 |
